# Triple Assessments of Atherosclerosis in Patients With Heterozygous Familial Hypercholesterolemia

**DOI:** 10.1016/j.jacasi.2025.04.011

**Published:** 2025-07-08

**Authors:** Hayato Tada, Nobuko Kojima, Kan Yamagami, Akihiro Nomura, Atsushi Nohara, Soichiro Usui, Kenji Sakata, Masa-aki Kawashiri, Masayuki Takamura

**Affiliations:** a Department of Cardiovascular Medicine, Graduate School of Medical Sciences, Kanazawa University, Kanazawa, Japan; b Department of Clinical Genetics, Ishikawa Prefectural Central Hospital, Kanazawa, Japan; c Department of Internal Medicine, Kaga Medical Center, Kaga, Japan

**Keywords:** carotid plaque, coronary calcium score, coronary plaque, familial hypercholesterolemia, LDL cholesterol

## Abstract

**Background:**

Data on the appropriate timing and impact of atherosclerosis assessment in patients with heterozygous familial hypercholesterolemia (HeFH) are limited.

**Objectives:**

The authors aimed to determine when atherosclerotic changes occur and the utility of triple assessments of carotid plaque, coronary plaque, and coronary artery calcium (CAC) in patients with HeFH.

**Methods:**

Data from patients with HeFH in the primary prevention setting admitted to Kanazawa University Hospital between 2000 and 2020 who underwent triple atherosclerosis assessment and were followed up were retrospectively reviewed (n = 622, male = 306, mean age = 54 ± 13 years). Risk factors for coronary heart disease events were determined using the Cox proportional hazard model. Carotid plaque, coronary plaque, and CAC scores were plotted against age.

**Results:**

We found that the age was independently associated with coronary heart disease events. Regression equations of carotid plaque, coronary plaque, and CAC scores against age were Y = 0.12X – 2.07 (β coefficient = 0.12 [95% CI: 0.09-0.15]; r^2^ = 0.12), Y = 0.36X – 9.30 (β coefficient = 0.36 [95% CI: 0.26-0.46]; r^2^ = 0.14), and Y = 2.48X – 77.23 (β coefficient = 0.07 [95% CI: 0.04-0.10]; r^2^ = 0.23) in men and Y = 0.12X – 3.60 (β coefficient = 0.12 [95% CI: 0.08-0.16]; r^2^ = 0.18), Y = 0.33X – 11.75 (β coefficient = 0.33 [95% CI: 0.29-0.37]; r^2^ = 0.17), and Y = 2.23X – 89.47 (β coefficient = 0.09 [95% CI: 0.06-0.12]; r^2^ = 0.34) in women, respectively. Significant differences of cardiovascular events were observed among the groups according to atherosclerotic burden.

**Conclusions:**

On average, carotid plaque, coronary plaque, and CAC may develop at ages 17, 26, and 31 years in male patients and 30, 36, and 40 years in female patients with HeFH, respectively, based on regression equations. Furthermore, triple assessments help in risk stratification.

Patients with familial hypercholesterolemia (FH) caused by pathogenic sequence variations in the low-density lipoprotein receptor (*LDLR*) or its associated genes, including apolipoprotein B (*APOB*), proprotein convertase subtilisin/kexin type 9 (*PCSK9*), and low-density lipoprotein receptor adaptor protein 1 (*LDLRAP1*), are at extremely high risk for coronary heart diseases (CHDs) caused by chronic exposure to elevated low-density lipoprotein (LDL) cholesterol.^1^, ^2^, ^3^ Guidelines around the world are now recommending starting treatment at 8 to 10 years^4^, ^5^, ^6^ based on observations suggesting that early intervention could lead to significantly better prognosis.^7^^,^^8^ Although we have come to understand that we need to start treating patients with FH in childhood, data on the development of atherosclerosis in patients with FH are limited. Therefore, there is no clear indication for atherosclerosis assessments for patients with FH, especially regarding the appropriate timing of assessments. Although there are several imaging modalities, including carotid ultrasound and coronary computed tomography angiography (CTA), that can assess atherosclerosis less invasively, there is no prior data on the impact of combined assessments. Therefore, we aimed to determine when atherosclerotic changes occur and the utility of triple assessments of carotid plaque, coronary plaque, and coronary artery calcium (CAC) in patients with heterozygous familial hypercholesterolemia (HeFH) in the primary prevention setting.

## Methods

### Study population

We analyzed data from 932 patients diagnosed with HeFH aged 15 years or older using the Japan Atherosclerosis Society (JAS) 2022 criteria^6^^,^^9^ who were admitted to Kanazawa University Hospital between 2000 and 2020 and underwent carotid ultrasound and coronary CTA. The 3 major elements of the clinical criteria are as follows: 1) hyper LDL cholesterol (LDL cholesterol ≥180 mg/dL); 2) tendon or cutaneous xanthomas; and 3) family history of FH or premature CAD (first-degree relatives). When a patient meets 2 or more criteria, he or she is diagnosed as having definite FH. In our institute, carotid ultrasound is routinely performed for any patients with HeFH at the initial assessment, and then coronary CTA is strongly recommended for patients with any carotid plaque. Coronary CTA is also recommended for patients with any chest discomfort in cases with HeFH. A total of 125 patients were excluded because of a history of coronary revascularization, 96 were excluded because of missing data, and 93 were excluded because they were lost to follow-up. Finally, 622 patients were included in this study (Supplemental Figure 1).

### Clinical data assessment

Hypertension was defined as systolic blood pressure ≥140 mm Hg, diastolic blood pressure ≥90 mm Hg, or antihypertensive medication use. We used the definition of diabetes by the Japan Diabetes Society.^10^ In brief, patients are diagnosed as diabetic type if they fulfill at least 1 of the criteria: 1) fasting blood glucose level ≥126 mg/dL; 2) nonfasting blood glucose level ≥200 mg/dL; 3) blood glucose level after 2 hours post–75 g oral glucose tolerance test ≥200 mg/dL; or 4) HbA1c level ≥6.5%. They will be diagnosed as diabetes if they fulfill at least 1 of the criteria of blood glucose level (1 to 3), and HbA1c level (4). The current smoking status of the patients was also considered. Using automated instruments, we enzymatically measured serum concentrations of triglycerides, high-density lipoprotein cholesterol, and total cholesterol. LDL cholesterol level was determined enzymatically when the triglyceride level was ≥400 mg/dL; otherwise, it was determined using the Friedewald formula. Lipid levels were determined at baseline before treatment and at follow-up. Achilles tendon thickenings were assessed by x-ray using the standard procedure at baseline. CHD events were defined as CHD-related death, unstable angina, myocardial infarction, or staged revascularization.

### Carotid plaque score assessment

Parameters for carotid ultrasound were measured using an Aplio carotid ultrasound machine (Toshiba Medical Systems) with a 7.5-MHz transducer by trained sonographers blinded to the clinical data. The carotid plaque score was calculated by summing the maximum carotid plaque thickness, defined as focal intimal thickening ≥1.1 mm, in each segment on both sides (a + b + c + contralateral plaque thickness in each segment on both sides), as previously described.^11^

### Coronary plaque score assessment

Coronary CTA was performed using a dual-source 64-slice system (Somatom Definition Flash; Siemens Medical System), as described in a previous study.^7^ Two experienced radiologists who were blinded to the clinical status evaluated all scans separately. Uninterpretable segments were scored as being the same as the most proximal interpretable segment. Discrepancies in assessment were resolved by consensus reading. Coronary CTA was performed according to the American Heart Association 15-segment classification. We assigned a score (0-5) to each of the 15 coronary artery segments according to the Society of Cardiovascular Computed Tomography guidelines (0 normal: absence of plaque and no luminal stenosis; 1 minimal: plaque with stenosis <25%; 2 mild: 25%-49% stenosis; 3 moderate: 50%-69% stenosis; 4 severe: 70%-99% stenosis; 5 occluded).^12^ We defined a coronary plaque score as the sum of the scores of all coronary artery segments.

### CAC assessment

The details were described in a previous study.^13^ The CAC score was assessed using the Agatston method with dedicated software (SYNAPSE VINCENT, Fuji Film Medical). When it was difficult to determine the exact location of calcified lesions, we referred to contrast-enhanced images.

### Strata by triple assessments of atherosclerosis

Based on the triple assessments described in the previous text (carotid plaque score, coronary plaque score, and CAC score), we divided the patients into 3 groups. Group 1: patients all of whose scores = 0, group 2: any scores ≥0 to ≤ median; and group 3: any scores ≥ median.

### Genetic analysis

We used next-generation sequencing to evaluate the genotypes. In brief, the coding regions of *APOB*, *LDLR*, *LDLRAP1*, and *PCSK9* were sequenced as previously described.^14^ Copy number variations in *LDLR* were also assessed, as previously described, using the eXome Hidden Markov Model.^15^ We used the standard American College of Medical Genetics and Genomics criteria (“pathogenic” or “likely pathogenic”) to determine whether genetic variants were pathogenic.^16^

### Ethical considerations

The Ethics Committee of Kanazawa University approved this study (2015-219). All procedures complied with the ethical standards of the Committee on Human Research (institutional and national), the laws and guidelines of Japan, and the Declaration of Helsinki (1975, revised in 2008). All study participants provided informed consent for genetic analysis.

### Statistical analysis

Normally distributed continuous variables are presented as mean ± SD. However, continuous variables that did not follow a normal distribution are presented as median (IQR). All categorical variables were compared using the Fisher exact test or the chi-square test, and the results are reported as numbers or percentages. For independent variables, the Student’s *t*-test was used to compare means of continuous variables, and the nonparametric Wilcoxon Mann-Whitney rank sum test was used to compare median values. For categorical variables, we performed the chi-square test or Fisher post hoc test as indicated. Regression equations were assessed to analyze correlations between age and score. We estimated the onset of carotid plaque, coronary plaque, and CAC development based on the regression equations between coronary and carotid plaques and age (x-intercepts). We used generalized additive model as a nonlinear model in this study. The significance of trends was assessed using the Cochran–Armitage or Jonckheere–Terpstra trend tests. Cox proportional hazard model was used to identify risk factors associated with CHD events adjusting age, sex, hypertension, diabetes, smoking, LDL cholesterol, and pathogenic variants. The proportional hazards assumption was investigated by plotting and visually survival probabilities vs time after follow-up. From baseline, Kaplan-Meier cumulative survival curves were generated to compare the time to first CHD event. R was used for all statistical analyses. *P* values of 0.05 were used to indicate statistical significance.

## Results

### Clinical characteristics

The clinical characteristics of the participants are presented in Table 1. The mean age of patients was 54 ± 13 years, and approximately 50% of them were men. At baseline, the median LDL cholesterol level was 229 mg/dL (Q1-Q3: 205-275 mg/dL). In total, 425 patients (68.3%) had a pathogenic FH variant. When we divided patients according to carotid plaque, coronary plaque, and CAC scores, we found 201 patients who were classified as group 1 (all score = 0), 205 patients were classified as group 2 (any score ≥0 to ≤ median), and 169 patients who were classified as group 3 (any scores ≥ median), and found significant trends in variables such as age, sex, diabetes, hypertension, smoking, total cholesterol, triglycerides, high-density lipoprotein cholesterol, baseline LDL cholesterol, follow-up LDL cholesterol, and Achilles tendon thickening. When we divided the patients by sex, we found similar trends in both sexes (Supplemental Tables 1 and 2).

**Table 1:** Characteristics of the Study Participants

	All (N = 622)	Group 1 All Scores = 0 (n = 201)	Group 2 Any Scores ≥0 to ≤ Median (n = 252)	Group 3 Any Score ≥ Median (n = 169)	*P* Value for Trend
Age, y	54 ± 13	42 ± 13	59 ± 13	64 ± 11	<0.0001
Male	306 (49.2)	78 (38.8)	125 (49.6)	103 (60.9)	<0.0001
Hypertension	200 (32.1)	29 (14.4)	101 (40.1)	70 (41.4)	<0.0001
Diabetes	64 (10.3)	11 (5.5)	30 (11.9)	23 (13.6)	<0.0001
Smoking	217 (34.9)	41 (20.4)	101 (40.1)	75 (44.4)	<0.0001
Total cholesterol, mg/dL	318 (286-360)	316 (281-362)	318 (284-362)	320 (284-391)	0.021
Triglycerides, mg/dL	130 (91-176)	120 (70-160)	132 (100-177)	141 (90-186)	<0.0001
HDL cholesterol, mg/dL	46 (39-56)	50 (42-60)	45 (39-55)	43 (33-53)	<0.0001
LDL cholesterol, at baseline, mg/dL	229 (205-275)	227 (201-275)	229 (213-262)	260 (213-314)	<0.0001
LDL cholesterol, at follow-up, mg/dL	108 (90-127)	111 (94-132)	102 (84-120)	108 (88-128)	0.33
FH pathogenic variants	425 (68.3)	140 (69.7)	168 (66.7)	117 (69.2)	0.55
Achilles tendon thickening, at baseline, mm	7.7 (6.6-9.1)	6.6 (5.9-7.3)	7.3 (6.4-8.2)	8.1 (7.0-9.8)	<0.0001

Values are mean ± SD, n (%), or median (Q1-Q3).

FH = familial hypercholesterolemia; HDL = high-density lipoprotein; LDL = low-density lipoprotein.

### Carotid plaque, coronary plaque, and CAC scores distribution by age

**Figure 1:**
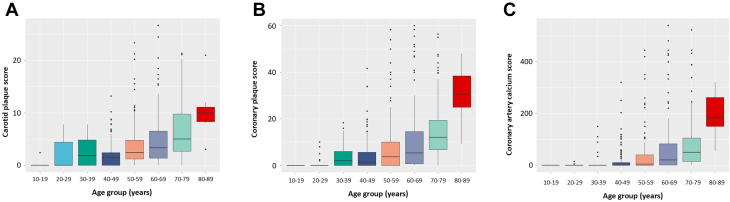
Carotid Plaque, Coronary Plaque, and Coronary Artery Calcium Scores by Age (A) Carotid plaque score: The x-axis represents age bins (by 10 years). The y-axis represents the carotid plaque score. (B) Coronary plaque score: The x-axis represents age bins (by 10 years). The y-axis represents the coronary plaque score. (C) Coronary artery calcium score: The x-axis represents age bins (by 10 years). The y-axis represents the coronary artery calcium score.

The carotid plaque, coronary plaque, and CAC scores appeared to increase with age (Figure 1). We found that none of the patients with HeFH had coronary plaque or CAC deposits in their teens, while some of the patients had a carotid plaque at that time.

### Correlation between score and age

**Figure 2:**
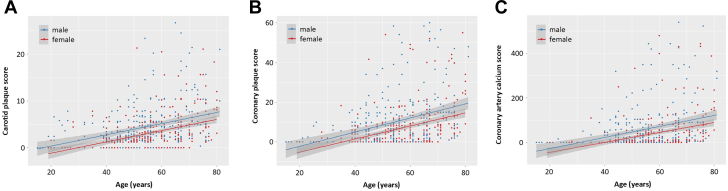
Correlations Between Plaque Scores and Age Using a Linear Model (A) Carotid plaque score: The x-axis represents age. The y-axis represents the carotid plaque score. (B) Coronary plaque score: The x-axis represents age. The y-axis represents the coronary plaque score. (C) Coronary artery calcium score: The x-axis represents age. The y-axis represents the coronary artery calcium score.

**Figure 3:**
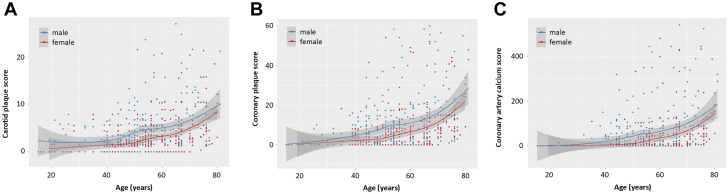
Correlations Between Plaque Scores and Age Using a Nonlinear Model (A) Carotid plaque score: The x-axis represents age. The y-axis represents the carotid plaque score. (B) Coronary plaque score: The x-axis represents age. The y-axis represents the coronary plaque score. (C) Coronary artery calcium score: The x-axis represents age. The y-axis represents the coronary artery calcium score.

When we plotted the carotid plaque, coronary plaque, and CAC scores against age in men and women, we found significant positive correlations between each score and age. When we used linear regression models, the regression equations of carotid plaque, coronary plaque, and coronary artery calcium (CAC) scores were Y = 0.12X – 2.07 (β coefficient = 0.12 [95% CI: 0.09-0.15]; r^2^ = 0.12), Y = 0.36X – 9.30 (β coefficient = 0.36 [95% CI: 0.26-0.46]; r^2^ = 0.14), and Y = 2.48X – 77.23 (β coefficient = 0.07 [95% CI: 0.04-0.10]; r^2^ = 0.23) in men and Y = 0.12X – 3.60 (β coefficient = 0.12 [95% CI: 0.08-0.16]; r^2^ = 0.18), Y = 0.33X – 11.75 (β coefficient = 0.33 [95% CI: 0.29-0.37]; r^2^ = 0.17), and Y = 2.23X – 89.47 (β coefficient = 0.09 [95% CI: 0.06-0.12]; r^2^ = 0.34) in women, respectively (Figure 2). Based on these equations, we can estimate that carotid plaque develops on average in 17 years in men and 30 years in women that coronary plaque develops on average in 26 years in men and 36 years in women, and CAC develops on average in 31 years in men and 40 years in women. Similar patterns were observed when we applied nonlinear models between age and score (Figure 3).

### LDL cholesterol by age

Lipid-lowering therapies at follow-up are illustrate in Supplemental Table 3. We also measured baseline LDL cholesterol level and follow-up LDL cholesterol level (after treatment) by age bins. We found that LDL cholesterol levels were more intensely reduced in older patients, although baseline LDL cholesterol levels were comparable (Supplemental Figure 2).

### Carotid plaque, coronary plaque, and CAC scores distribution

The carotid plaque, coronary plaque, and CAC scores were apparently higher in patients with CHD events (Supplemental Figure 3).

### Factors associated with CHD events

The median follow-up duration was 13.2 years (Q1-Q3: 9.8-18.4 years). We observed 132 CHD events during the follow-up period. Using the Cox proportional hazard model, we found that the following risk factors were significantly associated with CHD events: age (HR: 1.07; 95% CI: 1.02-1.12; *P <* 0.0001), male sex (HR: 1.76; 95% CI: 1.20-2.32; *P <* 0.0001), hypertension (HR: 2.10; 95% CI: 1.70-2.50; *P <* 0.0001), diabetes (HR: 1.48; 95% CI: 1.10-1.86; *P <* 0.0001), smoking (HR: 2.86; 95% CI: 1.80-3.92; *P <* 0.0001), LDL cholesterol (HR: 1.01; 95% CI: 1.00-1.02; *P =* 0.033, per 10 mg/dL), and the presence of pathogenic variants (HR: 1.64; 95% CI: 1.10-2.18; *P <* 0.0001) (Table 2).

**Table 2:** Factors Associated With CHD Events

	HR	95% CI	*P* Value
Age (per year)	1.07	1.02-1.12	<0.0001
Male (yes vs no)	1.76	1.20-2.32	<0.0001
Hypertension (yes vs no)	2.10	1.70-2.50	<0.0001
Diabetes (yes vs no)	1.48	1.10-1.86	<0.0001
Smoking (yes vs no)	2.86	1.80-3.92	<0.0001
LDL cholesterol (at baseline, per 10 mg/dL)	1.01	1.00-1.02	0.033
FH pathogenic variants (yes vs no)	1.64	1.10-2.18	<0.0001

Abbreviations as in Table 1.

### Prognosis by triple assessments of atherosclerotic score strata

**Figure 4:**
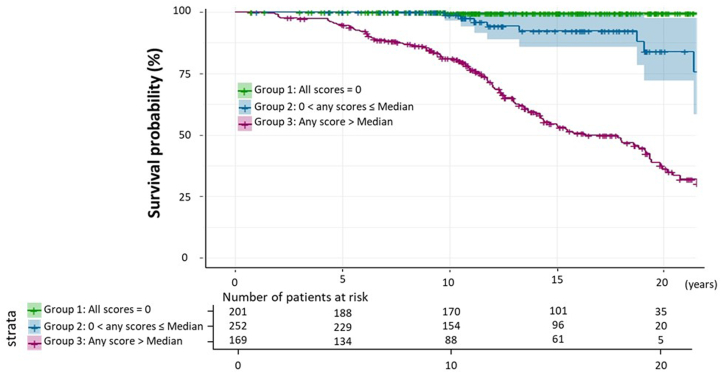
Kaplan-Meier Survival Curves Green indicates patients classified into group 1 (all scores = 0). Blue indicates patients classified into group 2 (others). Purple indicates patients classified into group 3 (any score > median).

Our assessment of the survival curve according to plaque and CAC scores strata revealed that patients with a score > median had the worst outcomes, and patients with all scores zero had the best outcome among the 3 groups (Figure 4). In fact, only 1 patient had unstable angina during the clinical course, probably caused by poor adherence to LDL-lowering therapy. The event rates per 1,000 person-years for group 1 (n = 201), group 2 (n = 252), and group 3 (n = 169) were 0.3 (95% CI: 0.2-0.4), 19.4 (95% CI: 10.2-28.6), and 65.9 (95% CI: 30.6-101.2), respectively.

## Discussion

In this study, we aimed to determine when atherosclerotic changes occur and to assess the utility of triple assessments of carotid plaque, coronary plaque, and CAC in patients with HeFH. We found they can develop on average at ages 17, 26, and 31 years and 30, 36, and 40 years of age in female patients with HeFH, respectively, and that triple assessments can help in risk stratification (Central Illustration).

**Central Illustration:**
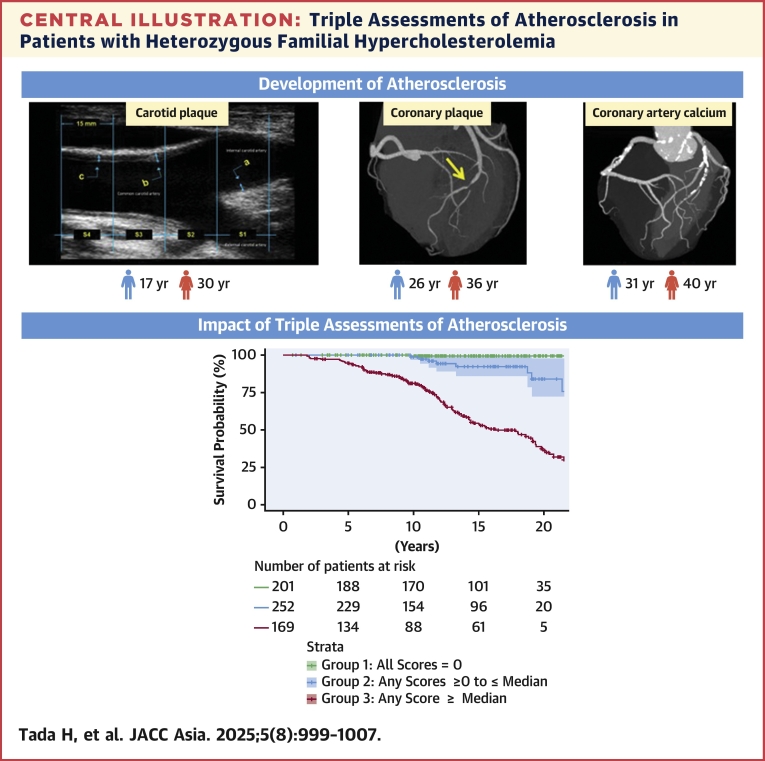
Triple Assessments of Atherosclerosis in Patients with Heterozygous Familial Hypercholesterolemia (Top) The ages of development of atherosclerosis. (Bottom) Impact of triple assessments of atherosclerosis.

Recent studies have suggested that the prevalence of HeFH is higher than previously considered.^17^ In fact, the prevalence of HeFH in Japan has been estimated to be around 1 in 300 general population,^18^ and this is also applicable to the United States and Europe.^19^^,^^20^ We know that the most important clinical manifestation of HeFH is early CHD. Dr Mabuchi previously reported that the incidence of myocardial infarction began to develop in patients with HeFH around the age of 30 years in men and 50 years in women.^21^ Furthermore, several observational studies have demonstrated that the initiation of LDL-lowering therapy during childhood was associated with a good prognosis.^7^^,^^8^ However, data on when atherosclerotic changes occur are limited. In other words, it is crucial to know when and how assess systemic atherosclerosis. There are several methods for assessing atherosclerosis, including carotid ultrasound, coronary CTA, and coronary angiogram (and intravascular imaging). These modalities have various strengths and limitations. For example, carotid ultrasound can noninvasively assess atherosclerosis, while carotid plaque burden may not reflect coronary artery disease. Conversely, coronary CTA can assess both coronary plaque and CAC plaque, but it requires contrast agents and radiation exposure. Therefore, we aimed to provide data on this issue among patients who underwent carotid ultrasound and coronary CTA. We found that carotid plaque developed before coronary plaque and CAC. Accordingly, we recommend carotid ultrasound assessment as early as possible in patients with HeFH, followed by coronary CTA if carotid plaques are detected. In fact, the similar trends were observed in non-FH individuals.^22^ The presence of carotid plaque has been shown as a good surrogate biomarker for systemic atherosclerosis, including coronary atherosclerosis. In fact, we are not fully sure why carotid plaque appear earlier than coronary artery in patients with HeFH. In this regard, no prior data exist assessing both plaque simultaneously in patients with HeFH. Further studies are needed to clarify if this phenomenon is universally observed in other ethnicities. Nevertheless, when patients with HeFH have a moderate amount of plaque or CAC, intensifying LDL-lowering therapy is recommended. Moreover, based on our findings that both coronary plaque and CAC begin developing after age 20 years, we recommend performing coronary CTA at age 20 years, even in patients without carotid plaque. On the other hand, we also found that a substantial proportions of patients with HeFH exhibited no atherosclerosis in coronary artery (eg, CAC = 0) despite their extreme high risk, which is consistent with the previous report.^23^ We believe that there are still many factors contributing to their risk for atherosclerosis other than HeFH.

### Study limitations

First, the single-center retrospective design may limit the generalizability of our findings to other patient populations. However, to our knowledge, this is the first study assessing this matter using multiple imaging modalities. Further studies investigating European and other ethnicities can refer to our results, given that most physicians are interested in racial differences. Second, we were unable to account for treatment discontinuations or modifications during follow-up, which may have influenced our results. Third, some patients were excluded from the analysis because of missing data or because they were lost to follow-up, which could have influenced our results. Fourth, we estimated the correlation coefficient under the assumption of linear regression, which may not apply to the development of atherosclerosis. Fifth, the timing of imaging, including carotid ultrasound and computed tomography, was not systematically determined in this study. Consequently, some assessments were obtained after the introduction of LDL-lowering therapies, which may have influenced our results. Finally, we did not account for traditional risk factors, such as diabetes, hypertension, and smoking, that were significantly collated with age in our equations. We believe that it is still quite important to control them among individuals with HeFH to prevent atherosclerosis.

## Conclusions

On average, carotid plaque, coronary plaque, and CAC may develop at the ages of 17, 26, and 31 in male patients and 30, 36, and 40 in female patients with HeFH, respectively. Triple assessment helps in risk stratification.

### Data Availability Statement

Requests for access to the data sets should be directed to Dr Hayato Tada.

## Funding Support and Author Disclosures

This work was supported by JSPS KAKENHI (20H03927, 21H03179, and 22H03330); a grant from the Ministry of Health, Labor, and Welfare of Japan (Science Research Grant for Research on Rare and Intractable Diseases) and the Japanese Circulation Society (project for genome analysis in cardiovascular diseases); and the Japan Agency for Medical Research and Development (20314864 and 22672854) to Dr Tada. The authors have reported that they have no relationships relevant to the contents of this paper to disclose.
