# Supplementary material for: Triple Assessments of Atherosclerosis in Patients With Heterozygous Familial Hypercholesterolemia
Source: JACC Asia. 2025 Jul 8;5(8):999–1007. doi: 10.1016/j.jacasi.2025.04.011 (PMC12426841; doi:10.1016/j.jacasi.2025.04.011)
Supplement: Supplemental Figures 1-3 and Supplemental Tables 1-3 [file mmc1.docx]

Supplemental Material

**Supplemental Figure 1. Strengthening the reporting of observational studies in epidemiology flow diagram of study selection**

**
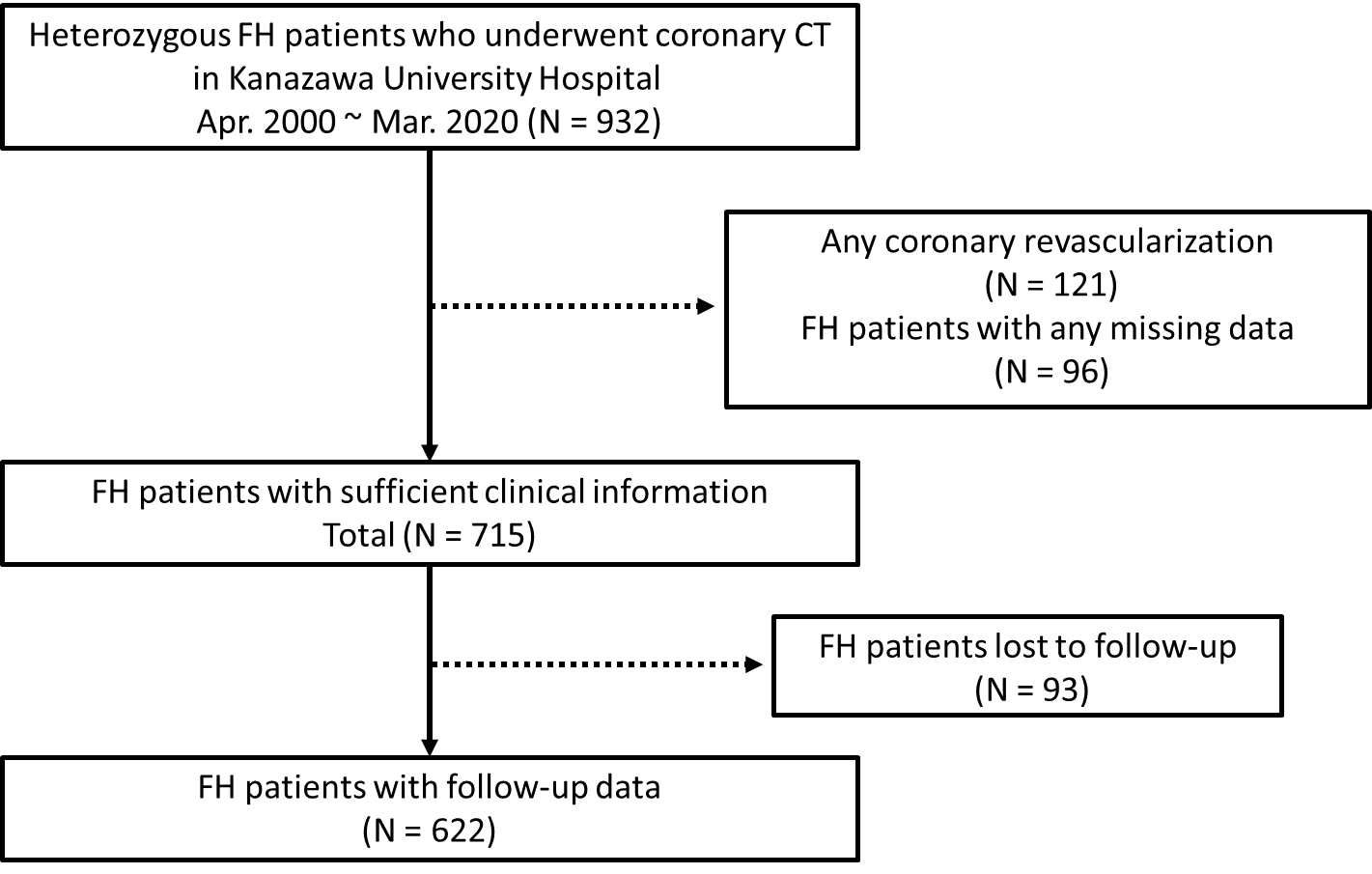
**

This flow delineates the selection and exclusion criteria for patient enrollment in this study. FH, familial hypercholesterolemia; CT, computed tomography

**Supplemental Figure 2. LDL cholesterol level before and after the treatment by age**

**
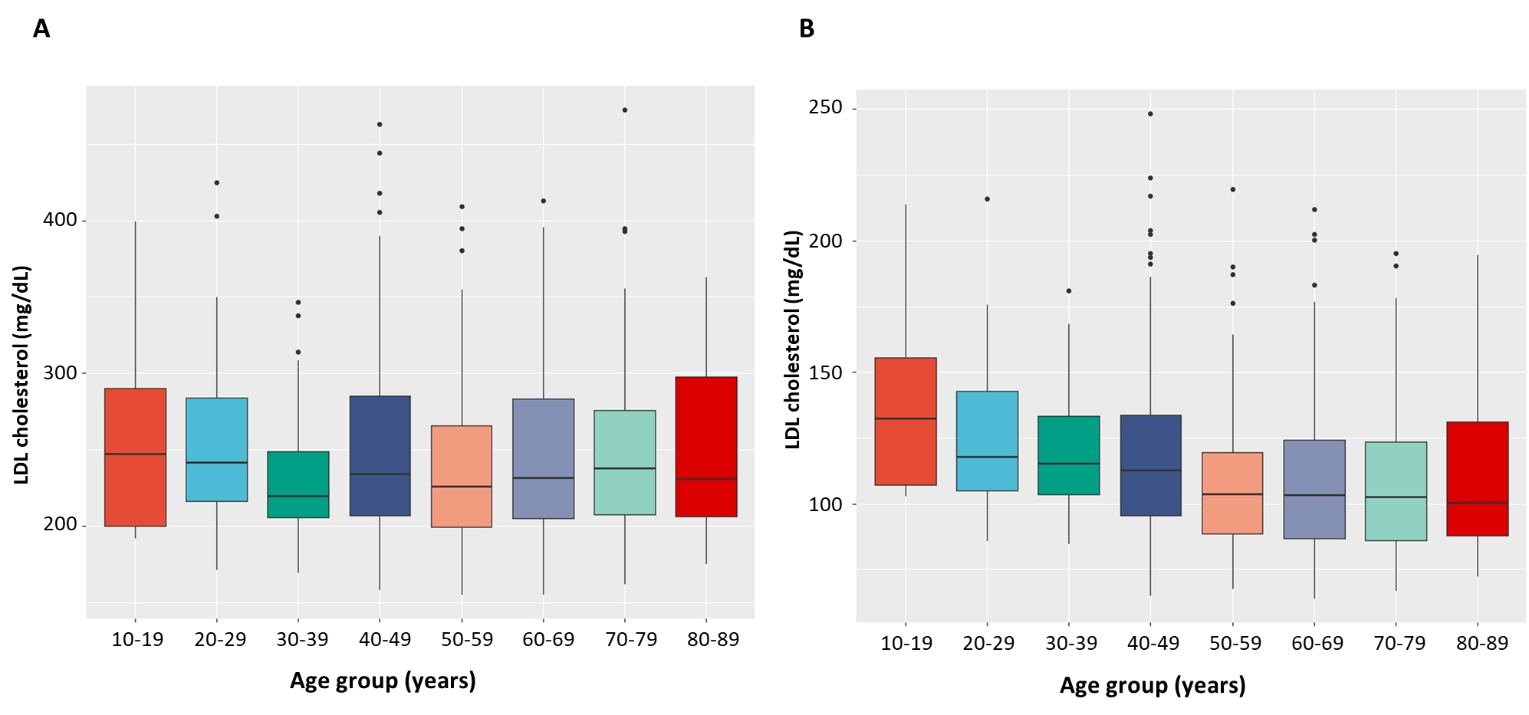
**

**Supplemental Figure 3. Carotid plaque, coronary plaque, and CAC scores distribution**

**
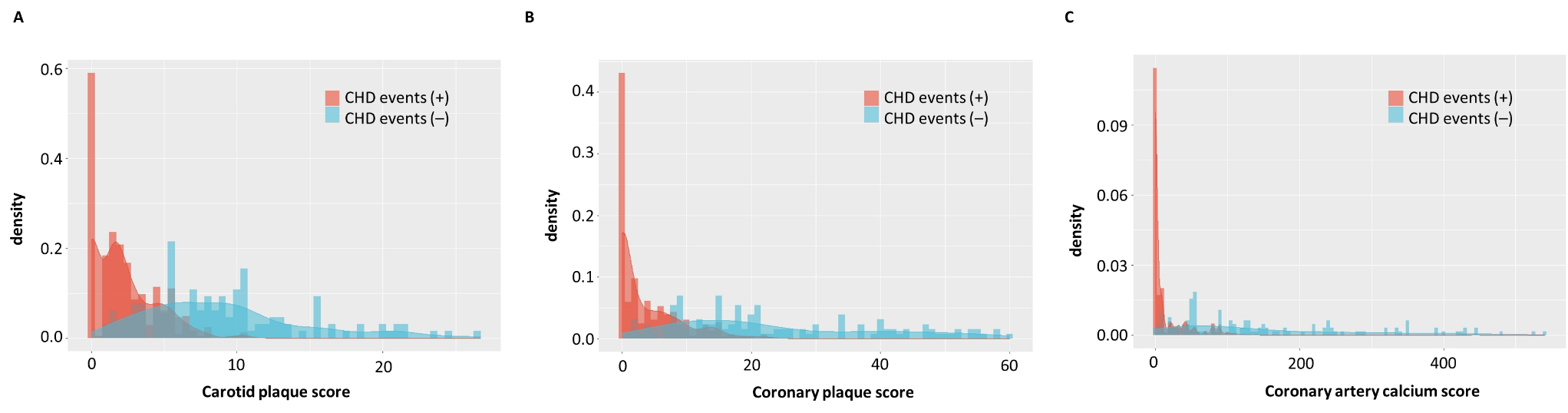
**

**Supplemental Table 1. Characteristics** **of the study participants (male)**

| Variables | All | Group 1 | Group 2 | Group 3 | *P*-value |
| --- | --- | --- | --- | --- | --- |
|  |  | All scores = 0 | 0 ≤ any scores ≤ Median | Any score > median |  |
|  | (N = 306) | (N = 83) | (N = 122) | (N = 101) | for trend |
| Age (years) | 51 ± 14 | 39 ± 13 | 55 ± 13 | 62 ± 11 | <0.0001 |
| Hypertension (%) | 98 (32.0%) | 13 (15.7%) | 47 (38.5%) | 38 (37.6%) | <0.0001 |
| Diabetes (%) | 30 (9.8%) | 5 (6.0%) | 12 (9.8%) | 13 (12.9%) | <0.0001 |
| Smoking (%) | 147 (48.0%) | 26 (8.5%) | 56 (45.9%) | 65 (64.3%) | <0.0001 |
| Total cholesterol (mg/dL) | 316 [284–356] | 314 [279–359] | 316 [282–360] | 318 [280–388] | 0.033 |
| Triglycerides (mg/dL) | 137 [94–187] | 110 [66–150] | 134 [101–176] | 156 [106–206] | <0.0001 |
| HDL cholesterol (mg/dL) | 44 [37–54] | 50 [42–59] | 44 [38–54] | 41 [33–51] | <0.0001 |
| LDL cholesterol (at baseline, mg/dL) | 226 [202–273] | 219 [199–272] | 224 [209–260] | 250 [220–314] | <0.0001 |
| LDL cholesterol (at followup, mg/dL) | 106 [88–125] | 114 [96–130] | 101 [85–121] | 108 [90–130] | 0.33 |
| FH pathogenic variants (%) | 206 (67.3%) | 56 (18.3%) | 72 (59.0%) | 78 (77.2%) | 0.21 |
| Achilles tendon thickening (at baseline, mm) | 7.9 [6.8–9.4] | 6.9 [6.6–7.8] | 7.5 [6.8–8.6] | 8.4 [7.5–10.4] | <0.0001 |

FH, familial hypercholesterolemia.

**Supplemental Table 2. Characteristics of the study participants (female)**

| Variables | All | Group 1 | Group 2 | Group 3 | *P*-value |
| --- | --- | --- | --- | --- | --- |
|  |  | All scores = 0 | 0 ≤ any scores ≤ Median | Any score > median |  |
|  | (N = 316) | (N = 118) | (N = 130) | (N = 68) | for trend |
| Age (years) | 57 ± 13 | 48 ± 14 | 60 ± 13 | 67 ± 11 | <0.0001 |
| Hypertension (%) | 102 (32.3%) | 11 (9.3%) | 56 (43.1%) | 35 (51.4%) | <0.0001 |
| Diabetes (%) | 34 (10.8%) | 4 (3.4%) | 16 (12.3%) | 14 (20.6%) | <0.0001 |
| Smoking (%) | 70 (22.1%) | 12 (10.2%) | 30 (23.1%) | 28 (41.2%) | <0.0001 |
| Total cholesterol (mg/dL) | 320 [288–359] | 316 [277–351] | 321 [284–360] | 326 [281–386] | <0.0001 |
| Triglycerides (mg/dL) | 126 [92–182] | 108 [60–144] | 129 [106–174] | 140 [108–201] | <0.0001 |
| HDL cholesterol (mg/dL) | 48 [40–56] | 52 [44–60] | 47 [40–56] | 43 [36–52] | <0.0001 |
| LDL cholesterol (at baseline, mg/dL) | 231 [206–279] | 221 [204–276] | 230 [211–258] | 251 [224–316] | <0.0001 |
| LDL cholesterol (at followup, mg/dL) | 110 [90–133] | 118 [90–131] | 108 [87–120] | 105 [80–120] | 0.033 |
| FH pathogenic variants (%) | 219 (69.3%) | 74 (62.7%) | 86 (61.7%) | 59 (86.8%) | 0.18 |
| Achilles tendon thickening (at baseline, mm) | 7.5 [6.6–8.8] | 6.6 [6.3–7.6] | 7.3 [6.7–8.4] | 8.0 [7.3–9.4] | <0.0001 |

FH, familial hypercholesterolemia.

**Supplemental Table 3. Lipid-lowering therapies**

| Lipid-lowering therapies | All | CHD event (+) | CHD event (–) |
| --- | --- | --- | --- |
|  | (N = 622) | (N = 132) | (N = 490) |
| Statins (%) | 610 (98.1%) | 131 (99.2%) | 479 (97.8%) |
| Ezetimibe (%) | 387 (62.2%) | 92 (69.7%) | 295 (60.2%) |
| Colestimide (%) | 135 (21.7%) | 28 (21.2%) | 107 (21.8%) |
| Probcol (%) | 1 (0.2%) | 1 (0.8%) | 0 (0.0%) |
| PCSK9 inhibitor (%) | 32 (5.1%) | 10 (7.6%) | 22 (4.5%) |
| LDL apheresis (%) | 1 (0.2%) | 1 (0.8%) | 0 (0.0%) |
| Fibrates (%) | 5 (0.8%) | 2 (1.5%) | 3 (0.6%) |
| n-3 PUFAs (%) | 9 (1.4%) | 3 (2.3%) | 6 (1.2%) |

PCSK9: proprotein convertase subtilisin/kexin type 9, PUFA: polyunsaturated fatty acid
